# Coevolution and cross-infection patterns between viruses and their host methanogens in paddy soils

**DOI:** 10.1093/ismeco/ycaf088

**Published:** 2025-05-23

**Authors:** Xingjie Wu, Ye Liu, Zhibin He, Xi Zhou, Werner Liesack, Jingjing Peng

**Affiliations:** State Key Laboratory of Nutrient Use and Management, College of Resources and Environmental Sciences, National Academy of Agriculture Green Development, Key Laboratory of Plant-Soil Interactions, Ministry of Education, China Agricultural University, Beijing 100193, China; State Key Laboratory of Nutrient Use and Management, College of Resources and Environmental Sciences, National Academy of Agriculture Green Development, Key Laboratory of Plant-Soil Interactions, Ministry of Education, China Agricultural University, Beijing 100193, China; State Key Laboratory of Nutrient Use and Management, College of Resources and Environmental Sciences, National Academy of Agriculture Green Development, Key Laboratory of Plant-Soil Interactions, Ministry of Education, China Agricultural University, Beijing 100193, China; State Key Laboratory of Nutrient Use and Management, College of Resources and Environmental Sciences, National Academy of Agriculture Green Development, Key Laboratory of Plant-Soil Interactions, Ministry of Education, China Agricultural University, Beijing 100193, China; Max Planck Institute for Terrestrial Microbiology, Marburg 35043, Germany; State Key Laboratory of Nutrient Use and Management, College of Resources and Environmental Sciences, National Academy of Agriculture Green Development, Key Laboratory of Plant-Soil Interactions, Ministry of Education, China Agricultural University, Beijing 100193, China

**Keywords:** methanogens, virus, coevolution, CRISPR-Cas, methane

## Abstract

Methanogens play a critical role in global methane (CH_4_) emissions from rice paddy ecosystems. Through the integration of metagenomic analysis and meta-analysis, we constructed a CRISPR spacer database comprising 14 475 spacers derived from 351 methanogenic genomes. This enabled the identification of viruses targeting key methanogenic families prevalent in rice paddies, including *Methanosarcinaceae*, *Methanotrichaceae*, *Methanobacteriaceae*, *Methanocellaceae*, and *Methanomassiliicoccaceae*. We identified 419 virus–host linkages involving 56 methanogenic host species and 189 viruses, spanning the families *Straboviridae*, *Salasmaviridae*, *Kyanoviridae*, *Herelleviridae*, and *Demerecviridae*, along with 126 unclassified viral entities. These findings highlight a virome composition that is markedly distinct from those observed in gut environments. Cross-infection patterns were supported by the presence of specific viruses predicted to infect multiple closely related methanogenic species. Evidence for potential virus–host coevolution was observed in 24 viruses encoding anti-CRISPR proteins, likely facilitating evasion of host CRISPR-mediated immunity. Collectively, this study reveals a complex and dynamic network of virus–host interactions shaping methanogen communities in rice paddy ecosystems.

Methanogenic archaea, a polyphyletic assemblage of archaeal lineages, possess a unique metabolic capability—methanogenesis—that directly controls methane (CH_4_) emissions in rice paddy ecosystems [1–3]. Both methanogens, which have persisted on Earth for ~3.5 billion years, and viruses, dating back to around 3.8 billion years, represent some of the most ancient biological entities [4, 5]. Viral predators play a significant ecological role by influencing the population dynamics of carbon-cycling methanogens, particularly along environmental gradients such as permafrost thaw zones [6]. To date, only a single virus, the siphovirus MFTV1, has been isolated from a deep-sea methanogen (*Methanocaldococcus fervens* AG 86) in the Guaymas Basin using traditional cultivation-dependent methods [7]. However, recent studies have identified novel virus families associated with gut methanogens, indicating a broader diversity of methanogen-infecting viruses than previously thought [8]. Given the potential ecological application of methanogen-associated viruses to mitigate global warming, investigating the virome of methanogens in rice paddies should be considered a research priority [9].

Rice paddies are recognized as a significant anthropogenic source of CH_4_ emissions [10]. Within these environments, five methanogenic families (*Methanosarcinaceae*, *Methanotrichaceae*, *Methanobacteriaceae*, *Methanocellaceae*, and *Methanomassiliicoccaceae*) are commonly identified as keystone taxa contributing to CH_4_ production [11, 12]. Despite their ecological importance, the associated virome of these methanogenic lineages remains poorly known. To address this gap, we conducted a genome-resolved metagenomic survey of paddy soils collected from four geographically distinct sites in China: Changsha (106.540°N, 29.403°E), Chongqing (112.986°N, 28.256°E), Ningxia (106.243°N, 38.473°E), and Jiansanjiang (116.726°N, 39.827°E).

From these samples, we recovered 43 methanogen genomes, comprising 25 high-quality and 18 medium-quality metagenome-assembled genomes (MAGs), spanning all five aforementioned families (Supplementary Fig.  1). In addition, 308 MAGs from these methanogenic families were retrieved from public databases, resulting in a total of 351 representative genomes (Supplementary Fig. 1). Phylogenetic analysis of these MAGs revealed five distinct family-level clusters (Fig. 1A).

**Figure 1:**
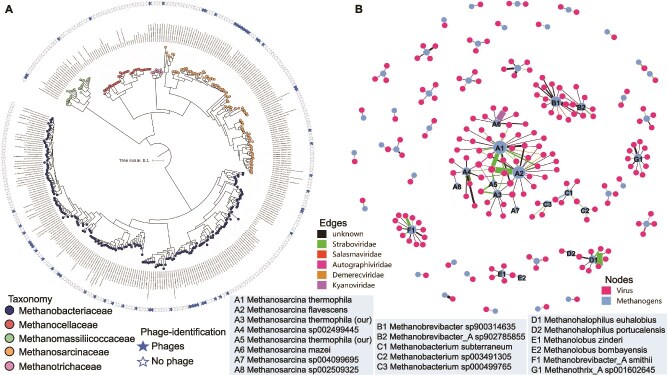
Phylogenetic tree of 351 methanogen genomes inferred using maximum likelihood analysis, based on a concatenated alignment of 122 universal archaeal marker genes (A). Solid asterisks indicate methanogens with viruses identified via the CRISPR-Cas system. The color of the circle dots represents different methanogen families: *Methanosarcinaceae*, *Methanotrichaceae*, *Methanobacteriaceae*, *Methanocellaceae*, and *Methanomassiliicoccaceae*. The label “no phage” denotes genomes for which no viral matches were identified in the IMG-VR4 database [15]. Panel (B) shows a co-occurrence network visualizing the host-virus interactions between methanogen genomes and their associated viromes. The network was constructed based on CRISPR-based host-virus linkages, with blue nodes representing methanogen hosts and red nodes representing methanogen-associated viruses. Edges indicate viral infections of methanogen hosts.

We then examined the presence of CRISPR-Cas systems across these genomes and identified Type I or Type III CRISPR-Cas loci in 191 methanogens, suggesting that these paddy-dominant lineages have evolved adaptive immune systems to counter viral invasion [13]. To further investigate virus-host interactions, we extracted CRISPR spacers from the 351 methanogen genomes, generating a database comprising 14 475 unique spacers (Supplementary Fig. 1). These spacers were subsequently used to search for matching protospacers within the IMG/VR v4 viral database [14], leading to the identification of 189 viral genomes that potentially infect 56 distinct methanogenic archaeal hosts (Fig. 1A).

These 189 viral genomes comprised seven complete genomes, 23 high-quality genomes, 47 medium-quality genomes, and 112 low-quality genomes (Supplementary Fig. 1). Taxonomic classification assigned the viruses to five known families: *Straboviridae* (*n* = 38), *Salasmaviridae* (*n* = 10), *Kyanoviridae* (*n* = 4), *Herelleviridae* (*n* = 4), and *Demerecviridae* (*n* = 3), while 123 viruses remained unclassified (Supplementary Fig. 1). The 189 viruses were affiliated with methanogen hosts from the following families: *Methanosarcinaceae* (18 of 77), and *Methanotrichaceae* (2 of 11), *Methanobacteriaceae* (33 of 94), *Methanocellaceae* (2 of 2), and *Methanomassiliicoccaceae* (2 of 5), (Fig. 1B; Supplementary Figs 2 and 3).

Host-virus associations were inferred using CRISPR spacer matches, yielding 419 linkages between the 189 viral genomes and 56 methanogen genomes (Fig. 1B, Supplementary Table 1). The distribution of host-virus linkages was as follows: *Methanosarcinaceae* (230 linkages; *Straboviridae*, *Demerecviridae*), *Methanotrichaceae* (17 linkages; *Zierdtviridae*, *Herelleviridae*, *Peduoviridae*), *Methanobacteriaceae* (160 linkages; *Straboviridae*, *Salasmaviridae*), *Methanocellaceae* (2 linkages; *Kyanoviridae*), and *Methanomassiliicoccaceae* (10 linkages; *Salasmaviridae*) (Fig. 1B). Among the viruses linked to *Methanosarcinaceae*, 18 were classified as *Straboviridae*, 3 as *Demerecviridae*, and 52 belonged to unclassified viruses, while those associated with *Methanobacteriaceae* were predominantly *Straboviridae* (*n* = 20), *Salasmaviridae* (*n* = 9), and unclassified viruses (*n* = 58). Notably, the viromes associated with paddy field methanogens exhibited distinct taxonomic compositions compared to those identified in gut-associated methanogens [8].

We observed distinct cross-infection patterns in host-virus interactions, wherein certain viruses were capable of infecting multiple closely related species within specific methanogen families (Fig. 1B). Within the *Methanosarcina* family, five major cross-infection modules (designated as Modules A–E) were identified (Fig. 1B). Module A comprised eight *Methanosarcina* species: *Methanosarcina thermophila* (A1), *Methanosarcina flavescens* (A2), *M. thermophila* (our MAGs, A3), *M.* sp. 002499445 (A4), *M. thermophila* (our MAGs, A5), *Methanosarcina mazei* (A6), *M.* sp. 004099695 (A7), and *M.* sp. 002509325 (A8). Nodes A3 and A5, corresponding to *M. thermophila*, were reconstructed from our metagenomic datasets, while A1, A2, and A6 represented cultured methanogen strains. Nodes A4 and A8 originated from fermentation-associated metagenomes, and A7 was recovered from sediment metagenomes. Module B consisted of two nodes, represented by two *Methanobrevibacter* strains: *M.* sp. 900 314 635 (B1) and *M.*_A sp. 902 785 855 (B2). Cross-infection events were also observed in Module C (3 nodes), Module D (2 nodes), and Module E (2 nodes), corresponding to the genera *Methanobacterium*, *Methanohalophilus*, and *Methanolobus*, respectively (Fig. 1B).

A proteome-scale phylogenetic analysis of the 189 methanogen-associated viruses, in comparison with 5420 reference double-stranded DNA (dsDNA) prokaryotic viral genomes, revealed the formation of two major phylogenetic clusters among the methanogen viruses (Supplementary Fig. 4). These two clusters exhibited close phylogenetic proximity. Of the viruses analyzed, 77 and 94 were associated with hosts from the families *Methanosarcinaceae* and *Methanobacteriaceae*, respectively (Fig. 2). Based on genome analysis, 100 viruses were classified as virulent and 79 as temperate phages (Fig. 2, Supplementary Table 2). Anti-CRISPR (Acr) systems were identified in 24 methanogen-associated viral genomes, corresponding to 7 viruses predicted to infect members of the *Methanosarcinaceae* and 17 infecting *Methanobacteriaceae* hosts (Fig. 2). These findings suggest that viruses infecting methanogens have evolved counter-defense mechanisms to evade host CRISPR-Cas immunity [16, 17].

**Figure 2:**
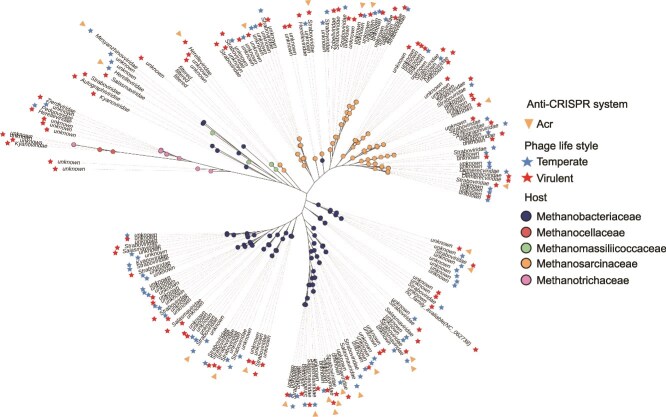
Proteome-scale phylogenetic tree of 189 methanogen viruses and their predicted hosts, categorized by the methanogen families *Methanosarcinaceae*, *Methanotrichaceae*, *Methanobacteriaceae*, *Methanocellaceae*, and *Methanomassiliicoccaceae*. The tree was constructed based on genome-wide sequence similarities, calculated using tBLASTx against 5420 reference dsDNA prokaryotic viral genomes. Blue and red asterisks indicate viruses predicted to exhibit temperate and virulent life cycles, respectively, as determined by the PhaTYP tool. Yellow triangles highlight the presence of anti-CRISPR proteins (Acrs) detected in the viral genomes.

Among the Acr-encoding viruses, 15 were classified as virulent and 9 as temperate. Taxonomic classification placed these viruses into the families *Herelleviridae* (*n* = 2), *Mesyanzhinovviridae* (*n* = 1), *Salasmaviridae* (*n* = 2), and *Straboviridae* (*n* = 6), with 11 viruses remaining unclassified. Structural annotation of virion proteins revealed components including major and minor capsid proteins, major and minor tail proteins, tail fibers, portal proteins, and baseplate structures (Supplementary Fig. 5). In addition, viral auxiliary metabolic genes (AMGs) were identified, including genes related to the 2-oxoglutarate (2OG)-Fe(II) oxygenase superfamily, glycosyltransferase family 2 (GT2) cellulose synthase, and phosphate starvation-inducible proteins. These AMGs are hypothesized to enhance host metabolic flexibility and fitness (Supplementary Fig. 6).

Collectively, our findings provide insights into the potential co-evolutionary dynamics between methanogenic archaea and their associated viromes. Evidence includes: (i) the presence of Type I and Type III CRISPR-Cas loci in 191 methanogen genomes, indicating host adaptive immunity; (ii) the identification of anti-CRISPR systems in 24 viral genomes, suggesting viral counter-defense strategies; and (iii) cross-infection of viruses among multiple methanogenic genera, including *Methanosarcina*, *Methanobrevibacter*, *Methanobacterium*, *Methanohalophilus*, and *Methanolobus*. These results illuminate the complexity of host–virus interactions in methanogens and suggest potential avenues for leveraging viruses to mitigate methane (CH_4_) emissions in paddy soil ecosystems.

## Supplementary Material

2025-05-21-Supplement_Materials_ycaf088

## Data Availability

The methanogen genomes obtained in this study are publicly available through NCBI under accession number PRJNA1131013. The associated viral genome sequences and relevant files are accessible via the following GitHub repository: https://github.com/Micropenglab/viruses-of-methanogens/.
