# Supplementary material for: Coevolution and cross-infection patterns between viruses and their host methanogens in paddy soils
Source: ISME Commun. 2025 May 23;5(1):ycaf088. doi: 10.1093/ismeco/ycaf088 (PMC12143468; doi:10.1093/ismeco/ycaf088)
Supplement: 2025-05-21-Supplement_Materials_ycaf088 [file 2025-05-21-supplement_materials_ycaf088.pdf]

Supplemental material

Coevolution and cross-infection patterns between viruses and their host methanogens in paddy soils

Xingjie Wu[1], Ye Liu[1], Zhibin He[1], Xi Zhou[1], Werner Liesack[2], Jingjing Peng[1*]

[1]State Key Laboratory of Nutrient Use and Management, College of Resources and Environmental Sciences, National Academy of Agriculture Green Development, Key Laboratory of Plant-Soil Interactions, Ministry of Education, China Agricultural University, Beijing 100193, China.

[2]Max Planck Institute for Terrestrial Microbiology, Marburg 35043, Germany.

*** Address correspondence to:** jingjing.peng@cau.edu.cn

Jingjing Peng: College of Resources and Environmental Sciences, China Agricultural University, Yuanmingyuan Xilu, Nr, 2. 100193, Beijing, China.

Supplementary Results and Discussion

We constructed a comprehensive CRISPR spacer database (n = 14,475 spacers) by extracting spacer sequences from CRISPR arrays across 351 methanogenic archaeal genomes, yielding an average of 41 spacers per genome (Supplementary Fig. 1). Using these spacers, we identified 419 virus–host linkages, connecting 56 methanogenic host genomes to 189 viruses from the families *Straboviridae*, *Salasmaviridae*, *Kyanoviridae*, *Herelleviridae*, and *Demerecviridae*. While CRISPR-based linkage methods provide relatively accurate host predictions, they have limitations, primarily reflecting historical rather than current virus–host interactions. This is especially relevant for methanogens lacking CRISPR-Cas systems. Furthermore, anti-CRISPR (Acr) genes were detected in 24 archaeal viral genomes, highlighting viral counter-defense strategies. Notably, several Acrs have been reported to exhibit broad-spectrum activity rather than strict host specificity [1], which further complicates interpretations of virus–host coevolution in methanogenic environments.

The presence of anti-CRISPR (Acr) systems was detected in members of the *Methanobacteriaceae* and in seven genomes belonging to the *Methanosarcinaceae*, but not in the *Methanotrichaceae*, *Methanomassiliicoccaceae*, or *Methanocellaceae* (Supplementary Figs. 6, 7). Viral auxiliary metabolic genes (AMGs), which may influence host metabolism and biogeochemical processes, were annotated using DRAM-V (Supplementary Fig. 6). AMGs were associated with antibiotic biosynthesis [2-oxoglutarate (2OG)-Fe(II) oxygenase superfamily], cellulose

metabolism (GT2 cellulose synthase), and phosphate starvation-inducible proteins, suggesting a potential role in enhancing host fitness and metabolic adaptability (Supplementary Fig. 6).

Significant genomic differences were observed between viruses encoding Acr genes (Acr-viruses) and those lacking them (non-Acr-viruses). Specifically, Acr-viruses exhibited significantly longer genome lengths and a higher number of predicted open reading frames (ORFs) compared to non-Acr-viruses ($P < 0.001$; Supplementary Fig. 8). Furthermore, prophages identified in methanogen genomes using VirSorter2 and CheckV were compared to corresponding viral genomes from the IMG/VR v4 database (Supplementary Fig. 9). No significant differences were observed in genome characteristics between prophages and IMG/VR viruses ($P > 0.05$).

Materials and methods

A collection of methanogen genome

The soil samples were collected from paddies soils from four provinces of Changsha (106.540°N, 29.403°E), Chongqing (112.986°N, 28.256°E), Ningxia (106.243°N, 38.473°E) and Heilongjiang (116.726°N, 39.827°E) in China. The anoxic microcosms were incubated with collected paddy soils at 15 ℃, 30 ℃ and 50 ℃ for 135 days to enrich the methanogens and perform the genome-resolved metagenomic sequencing. Soils DNA were extracted from 0.25 g incubated slurry

using the FastDNA Spin Kit for Soil according to the manufacture instructions. The metagenomic libraries were prepared with 1 μg purified DNA samples, and sequenced using the HiSeq 2500 with the 150 bp paired-end reads in Novegene Company (Tianjin, China). Raw metagenome sequences were quality filtered using Trimmomatic by removing the reads shorter than 50 bp and low-quality bases. The clean metagenomic reads were assembled into Contigs by the Megahit with the k-mer length increasing from 21 to 141 in steps of 20. Contigs longer than 1000 bp were binned into the metagenomic assembled genomes (MAGs) by using the MetaWRAP pipeline [2, 3].

To expand the datasets of *Methanosarcinaceae*, *Methanotrichaceae*, *Methanobacteriaceae*, *Methanocellaceae* and *Methanomassiliicoccaceae*, 308 methanogen genomes of these five families were downloaded from public database of NCBI and Genbank. The completenesss, contamination, genome size and GC content of methanogen genomes were estimated using Checkm (V1.1.2) [4]. The taxonomic information of methanogen genomes was achieved using the GTDB-Tk database (V1.3.0) [5]. The phylogenetic tree was constructed with GTDB-Tk by a concatenated alignment of 122 universal archaea marker gene and visualized in the i-Tol platform [6].

CRISPR-Cas based identification of virus associated with methanogens

CRISPR-Cas systems in methanogenic archaea genomes were detected using CRISPRCasTyper tools (https://github.com/Russel88/CRISPRCasTyper). Spacers

from validated CRISPR array to compare to viral contigs in the IMG/VR V4 database using BLASTN with parameters of evalue at e^{-5} [7]. The criteria for establishing host-virus linkages was adopted from a previous study [8]. Only viral sequences harboring protospacers and a maximum of one mismatch were referred methanogenic archaea genomes associated viruses which resulted in a total of 189 host-virus linkages. We referred to the datasets of 56 methanogenic archaea genomes associated with virus as the 'methanogen host', and the 189 viruses that infected the methanogens as 'methanogen virome'. The habitat of 56 methanogenic archaea genomes were searched in the NCBI based on the environments of collected metagenomic samples.

Classification of methanogenic archaea viruses

The quality of assembled virus contigs were assessed by the CheckV, which estimates completeness of viral contigs by comparing sequences with a systematic database that consist 76,262 complete viral genomes. According to the standard criteria, CheckV classified these 189 viruses as complete genomes, high-quality (> 90% completeness), medium-quality (50-90% completeness) and low-quality (<50% completeness) [9]. The proteome-scale phylogeny of 189 methanogen viruses was constructed based on genome-wide sequence similarities calculated by tBLASTx with 5420 reference dsDNA prokaryotic viral genomes in the latest version of VipTree tool [10]. The VipTree tool used reference viral sequences and associated host from the GenomeNet Virus-Host database [11]. The potential anti-CRISPR proteins (Acrs) of methanogenic archaea viruses were first predicted using the AcrFinder by the deep

learning algorithms (https://github.com/HaidYi/acrfinder) [12]. We applied the latest state-of-the-art virus classification tools of PhaGCN2 to perform taxonomic assignments of viral sequences [13]. The PhaGCN2 was reported to have greatly increased the precision of taxonomic classification in comparison to previous published tools, including vConTACT2, CAT and VPF-Class [13]. The network for virus-host linkages were visualized in the Gephi platform (https://gephi.org/). The virion proteins annotation in viral contigs was performed by using the the state-of-the-art classification model tools of PHaVIP (Phage VIrion Protein) (https://github.com/KennthShang/PhaVIP). The viral proteins were annotated using Diamond by blast against the NCBI non-redundant protein, KEGG and Pfam databases [14]. The virulent or temperate lifestyle of methanogen virome was predicted by using the PhaTYP (Phage TYPe prediction tool), which was confirmed to have the stablest performance when compared to the DeePhage, PHACTS, PhagePred and BACPHLIP [15].

Reference

1. Forsberg KJ. Anti-CRISPR discovery: using magnets to find needles in haystacks. J Mol Biol. 2023; 435: 167952.
2. Li D, Liu C, Luo R, Sadakane K, Lam T. MEGAHIT: an ultra-fast single-node solution for large and complex metagenomics assembly via succinct de Bruijn graph. Bioinfomatics. 2015; 31: 1674-6.

3. Uritskiy GV, DiRuggiero J, Taylor J. MetaWRAP—a flexible pipeline for genome-resolved metagenomic data analysis. Micorbiome. 2018; 6

4. Parks DH, Imelfort M, Skennerton CT, Hugenholtz P, Tyson GW. CheckM: assessing the quality of microbial genomes recovered from isolates, single cells, and metagenomes. Genome Res. 2015; 25: 1043-55.

5. Chaumeil PA, Mussig AJ, Hugenholtz P, Parks DH. GTDB-Tk: a toolkit to classify genomes with the Genome Taxonomy Database. Bioinfomatics. 2019

6. Letunic I, Bork P. Interactive tree of life (iTOL) v3: an online tool for the display and annotation of phylogenetic and other trees. Nucleic Acids Res. 2016; 44: W242-5.

7. Camargo AP, Nayfach S, Chen IA, Palaniappan K, Ratner A, Chu K, et al. IMG/VR v4: an expanded database of uncultivated virus genomes within a framework of extensive functional, taxonomic, and ecological metadata. Nucleic Acids Res. 2023; 51: D733-43.

8. Duan C, Liu Y, Liu Y, Liu L, Cai M, Zhang R, et al. Diversity of Bathyarchaeia viruses in metagenomes and virus-encoded CRISPR system components. ISME Communications 2024; 4: ycad11.

9. Nayfach S, Camargo AP, Schulz F, Eloe-Fadrosh E, Roux S, Kyrpides NC. CheckV assesses the quality and completeness of metagenome-assembled viral genomes. Nat Biotechnol. 2021; 39: 578-85.

10. Nishimura Y, Yoshida T, Kuronishi M, Uehara H, Ogata H, Goto S. ViPTree: the viral proteomic tree server. Bioinfomatics. 2017; 33: 2379-80.

11. Mihara T, Nishimura Y, Shimizu Y, Nishiyama H, Yoshikawa G, Uehara H, et al. Linking virus genomes with host taxonomy. Viruses Basel. 2016; 8: 66.

12. Yi H, Huang L, Yang B, Gomez J, Zhang H, Yin Y. AcrFinder: genome mining anti-CRISPR operons in prokaryotes and their viruses. Nucleic Acids Res. 2020; 48: W358-65.

13. Jiang JZ, Yuan WG, Shang J, Shi YH, Yang LL, Liu M, et al. Virus classification for viral genomic fragments using PhaGCN2. Brief Bioinform. 2023; 24

14. Mistry J, Chuguransky S, Williams L, Qureshi M, Salazar GA, Sonnhammer E, et al. Pfam: The protein families database in 2021. Nucleic Acids Res. 2021; 49: D412-9.

15. Shang J, Tang X, Sun Y. PhaTYP: predicting the lifestyle for bacteriophages using BERT. Brief Bioinform. 2023; 24

Supplemental Figures

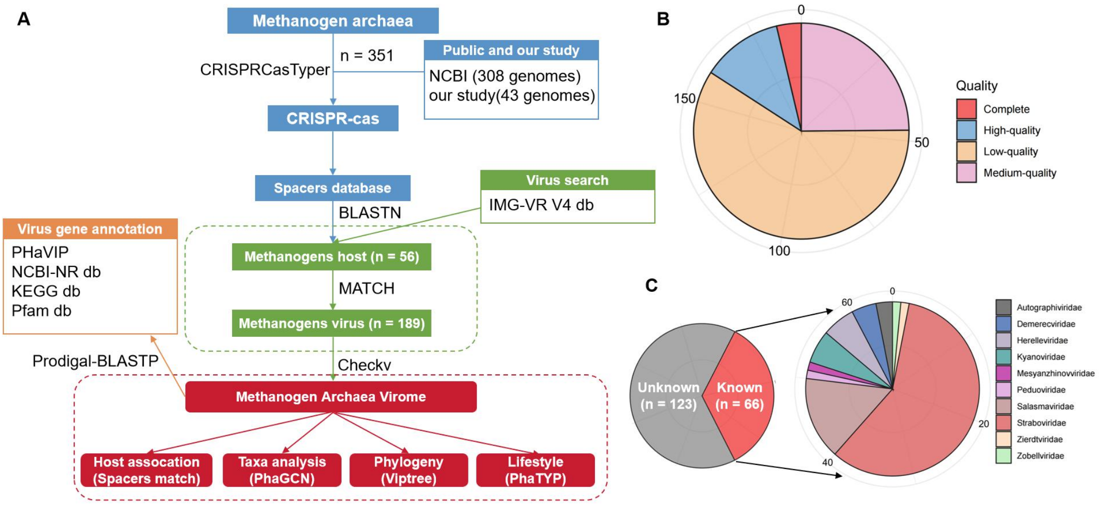

Fig. S1 Experimental design and characterization of methanogen viruses. (A) Overview of the analysis pipeline used for the identification of methanogen viruses, including CRISPR–Cas system detection, spacer database construction, and virus identification and classification across 351 methanogenic archaeal genomes. (B) Quality assessment of the identified viral genomes using CheckV, indicating the distribution of genome completeness. (C) Taxonomic classification of the methanogen virome at the family level.

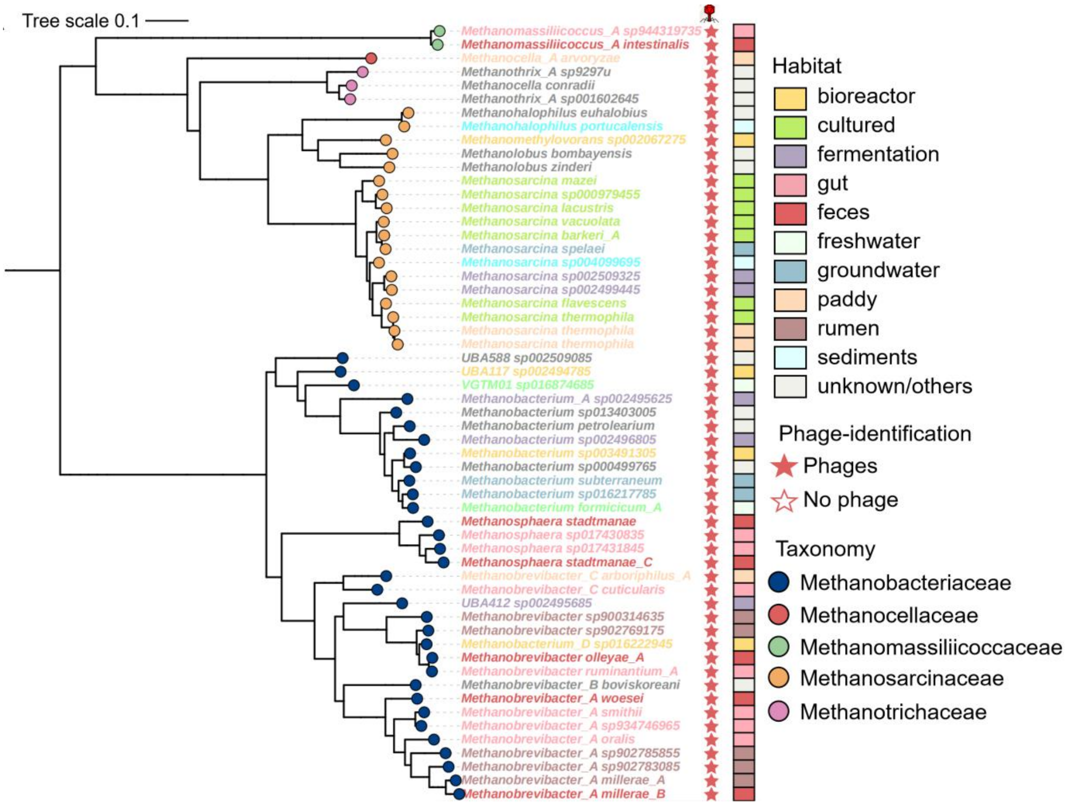

Fig. S2 Phylogenetic analysis of viruses infecting methanogens. Maximum-likelihood phylogenetic tree constructed from a concatenated alignment of 122 universal archaeal marker genes, representing viruses targeting 56 methanogenic archaeal genomes from diverse habitats. The tree was visualized using the iTOL platform. Asterisks denote host genomes in which viral infections were detected.

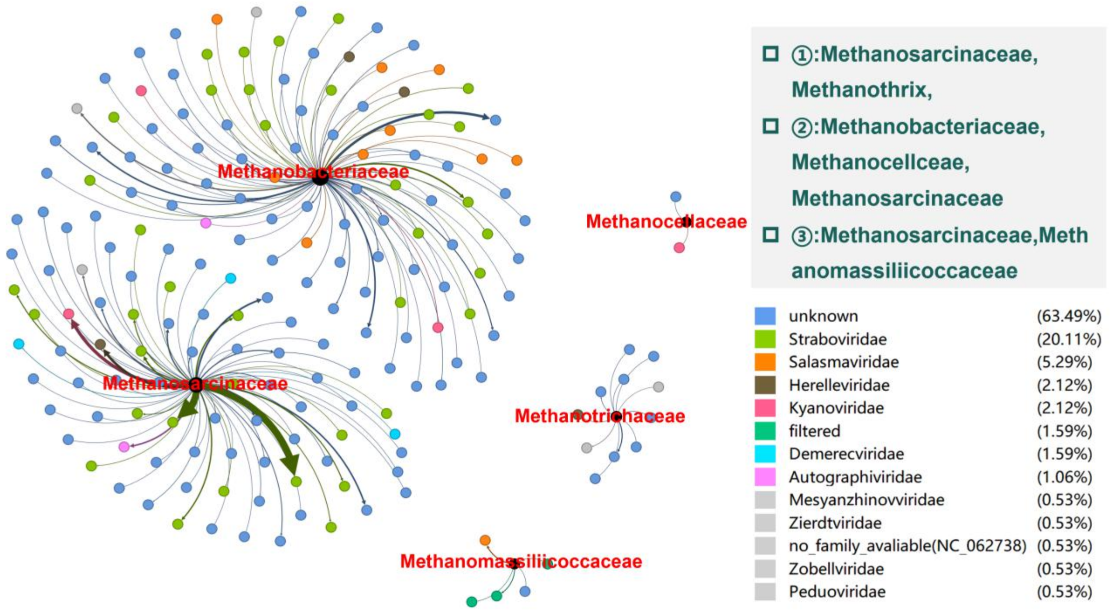

Fig. S3 Viral-host network of the methanogen virome. Network visualization of viral associations with methanogenic archaeal families, including *Methanosarcinaceae*, *Methanothrix*, *Methanobacteriaceae*, *Methanocellaceae*, and *Methanomassiliicoccaceae*.

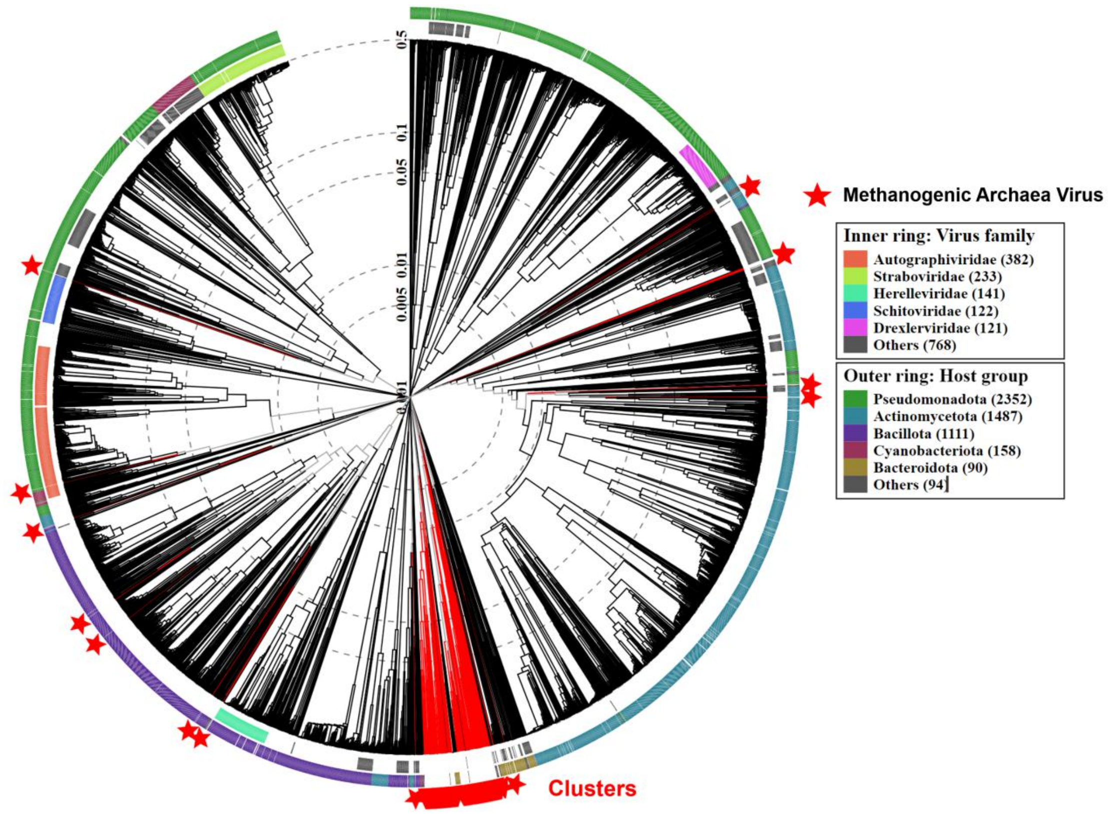

Fig. S4 Phylogenetic placement of 189 methanogen-associated viruses within a comprehensive proteome-scale phylogenetic tree comprising 5,420 reference dsDNA prokaryotic viral genomes from the GenomeNet Virus-Host database. Red asterisks indicate the positions of methanogen-associated viral genomes.

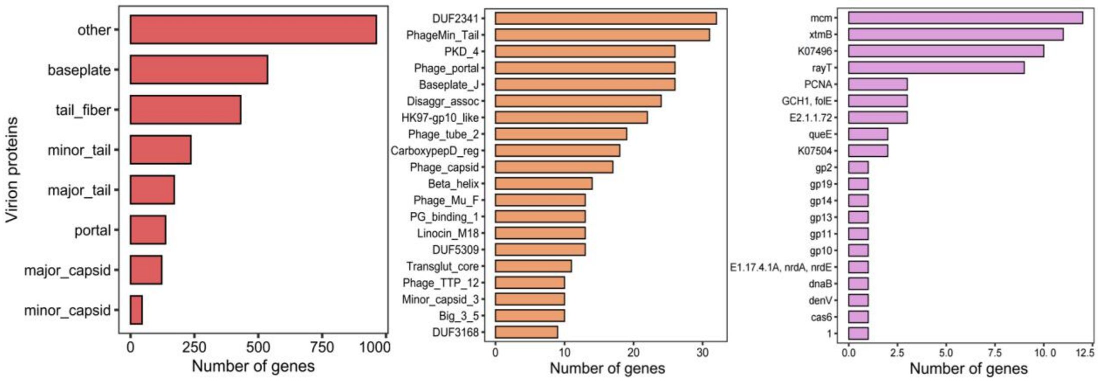

Fig. S5 Functional annotation of viral proteins derived from methanogen-associated viromes using three databases: PHaVIP (red bars), Pfam (yellow bars), and KEGG (purple bars).

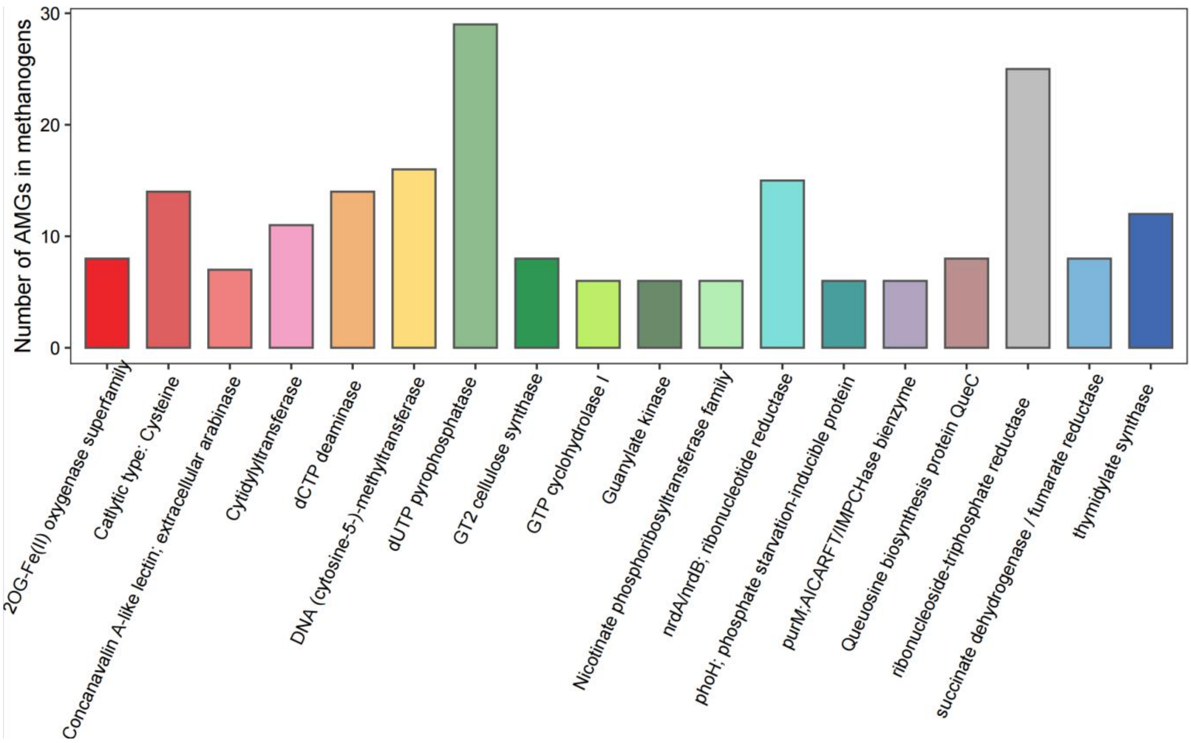

Fig. S6 Auxiliary metabolic genes (AMGs) identified in methanogen-associated viromes using the DRAM-V pipeline.

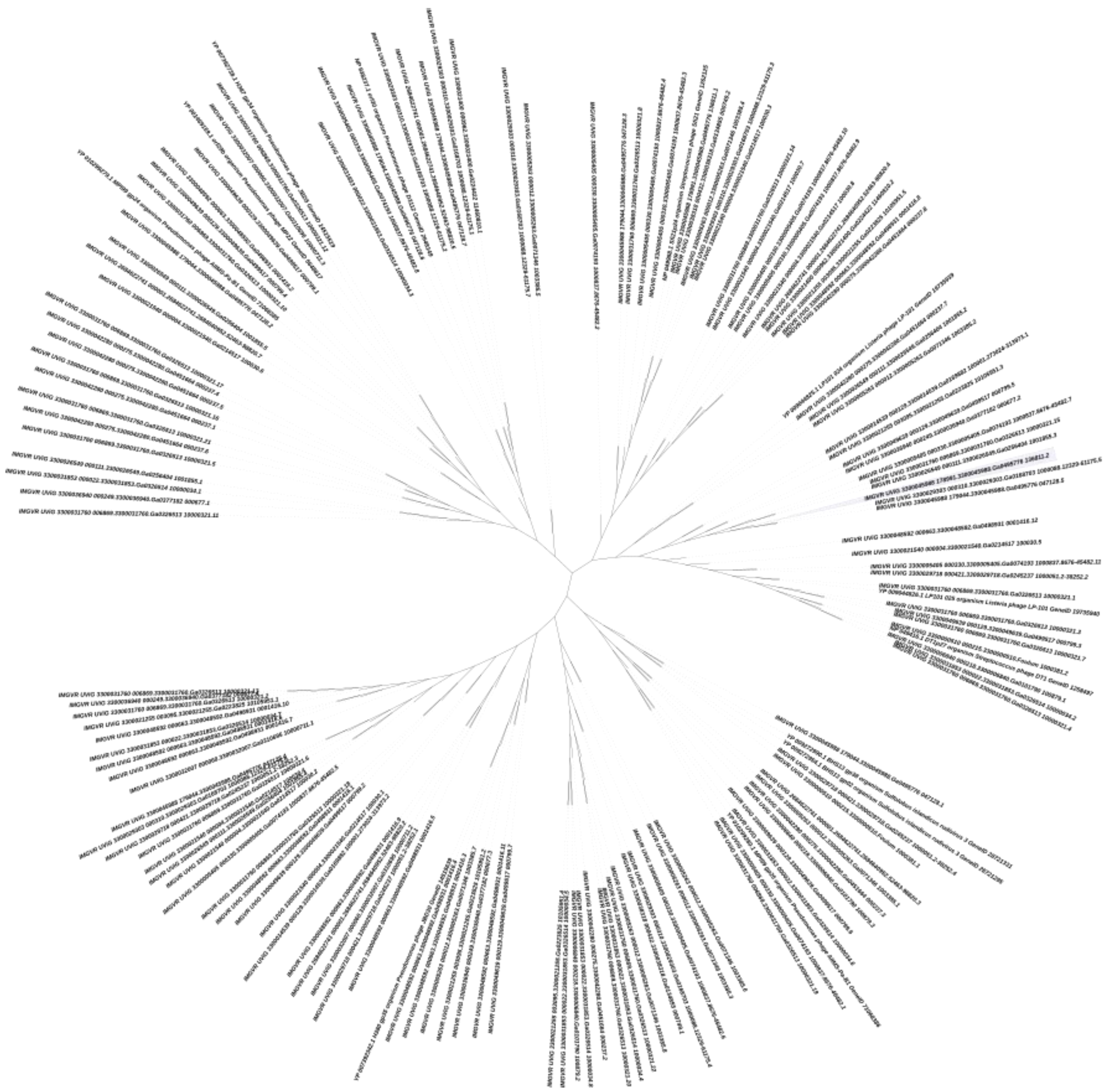

Fig. S7 Phylogenetic distribution of anti-CRISPR (Acr) proteins identified in methanogen-associated archaeal viruses using AcrFinder.

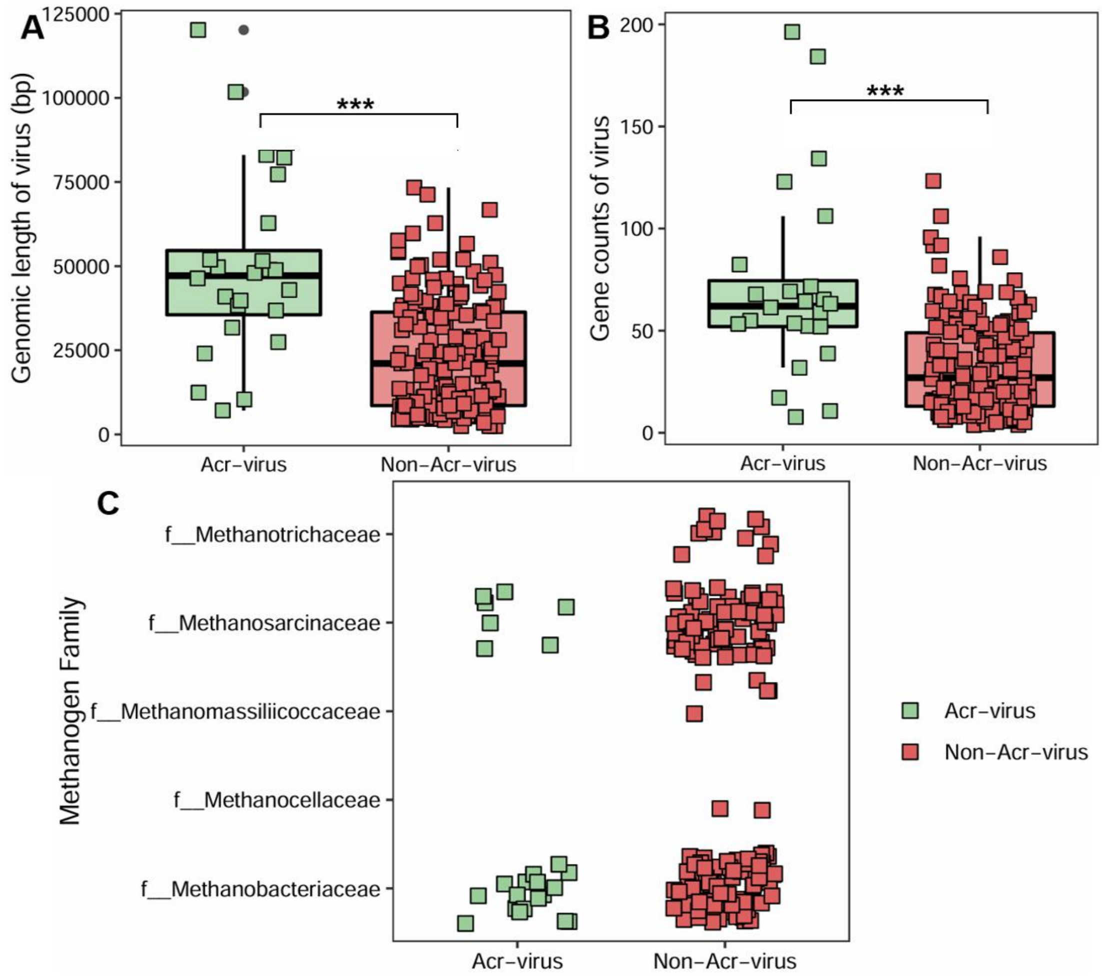

Fig. S8 (A) Comparison of genomic lengths and (B) number of infected host species between methanogen viruses encoding anti-CRISPR (Acr) proteins (Acr-virus) and those lacking them (Non-Acr-virus) (C). Taxonomic distribution of viral host families infected by Acr-viruses and Non-Acr-viruses. Asterisks (***) indicate statistically significant differences between groups ($P < 0.001$).

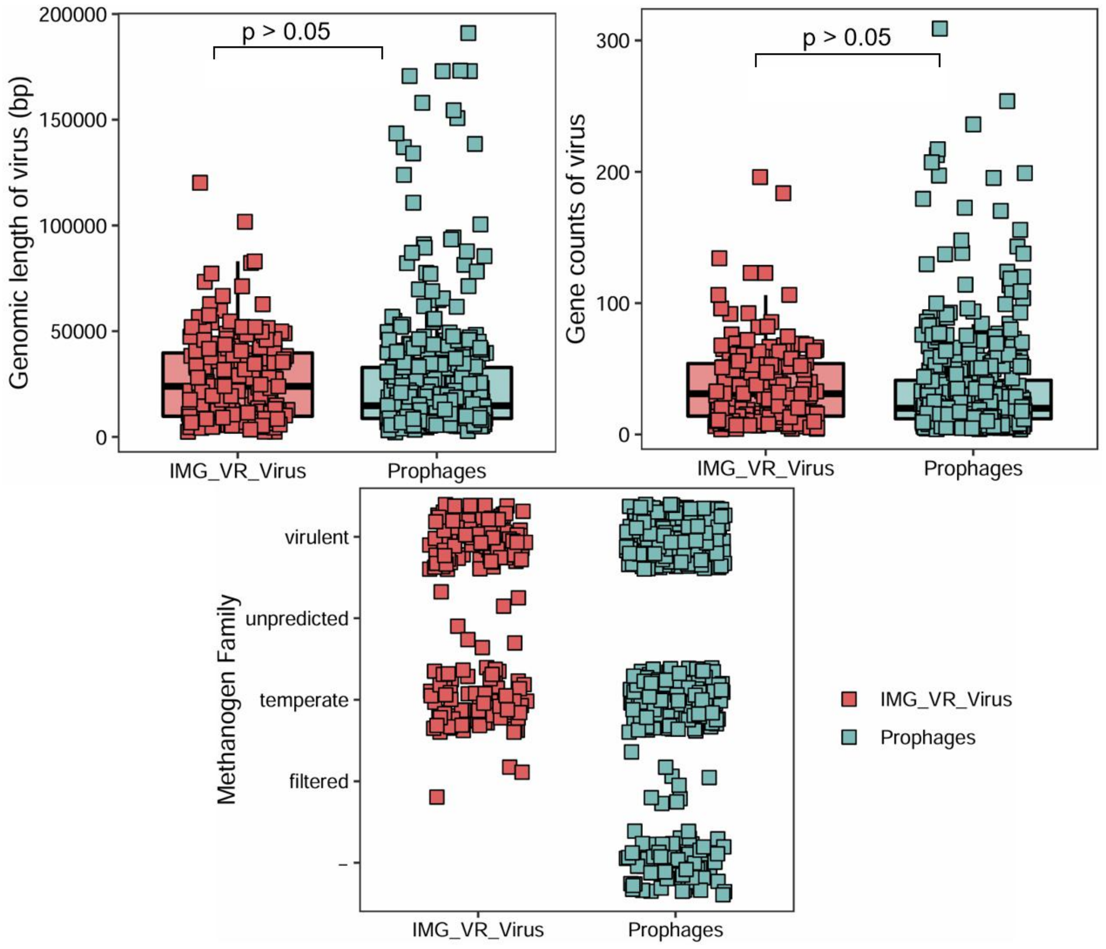

Fig. S9 Comparison of genomic length, number of infected host species, and lifestyle between methanogen viruses from the IMG/VR database and prophages. Prophages were identified within methanogen genomes using VirSorter2 and CheckV.

Supplemental Tables

Table S1 Detailed information on seven complete viral genomes targeting methanogens

Virus ID	Length	Genes	Completeness	Contamination	Source
Complete viral genome 1	59736	86	100	0	IMG-VR
Complete viral genome 2	45456	68	100	0	IMG-VR
Complete viral genome 3	38346	42	100	0	IMG-VR
Complete viral genome 4	38648	55	100	0	IMG-VR
Complete viral genome 5	50364	64	100	0	IMG-VR
Complete viral genome 6	46452	59	100	0	IMG-VR
Complete viral genome 7	56676	66	100	0	IMG-VR

Table S2 Viral characteristics associated with methanogens: Lifestyle and Abundance

**Methanogen Family**	**Lifestyle**	**Number of Viruses**
f__*Methanomassiliicoccaceae*	unpredicted	3
f__*Methanobacteriaceae*	temperate	48
f__*Methanomassiliicoccaceae*	temperate	1
f__*Methanosarcinaceae*	temperate	28
f__*Methanotrichaceae*	temperate	2
f__*Methanobacteriaceae*	unpredicted	1
f__*Methanosarcinaceae*	unpredicted	5
f__*Methanotrichaceae*	unpredicted	1
f__*Methanobacteriaceae*	virulent	45
f__*Methanocellaceae*	virulent	2
f__*Methanomassiliicoccaceae*	virulent	1
f__*Methanosarcinaceae*	virulent	44
f__*Methanotrichaceae*	virulent	8
